# Supplementary material for: A comprehensive AI model development framework for consistent Gleason grading
Source: Commun Med (Lond). 2024 May 9;4:84. doi: 10.1038/s43856-024-00502-1 (PMC11082180; doi:10.1038/s43856-024-00502-1)
Supplement: Supplementary file 2 — Supplementary Information [file 43856_2024_502_MOESM2_ESM.pdf]

A Comprehensive AI Model Development Framework for Consistent Gleason Grading

Supplementary Material

Supplementary Methods **2**

Supplementary Tables **8**

Supplementary Figures **10**

Supplementary Notes **16**

Supplementary Methods

A!MagQC: A quantitative digital pathology image quality control solution

With the increasing use of digital pathology, vast amounts of data are generated on a daily basis. However, there are common quality issues (as shown in Supplementary **Figure 1a**), and visually assessing image quality has become a tedious and heavy workload for researchers. While perceptual image quality estimators based on perception-based image quality evaluators (PIQE) can calculate a no-reference image quality score, this approach has proven to be less effective for histological images. Although some tools have been developed previously for histological images, they have been limited to evaluating only Haematoxylin and Eosin (H&E) images.

To address this gap, A!MagQC was developed to provide fully automated quality control for any histologically relevant imaging modality, including Haematoxylin and Eosin (H&E), Immunohistochemistry (IHC), and Multiplexed Fluorescence (MF). The software automatically detects the image size (magnification) and type from the metadata of each image file. The user interface is shown in **Supplementary Figure 1b**.

The first step in assessing the quality of tissue images is to detect the tissue and separate it from the background. This is achieved by applying adaptive thresholding. To evaluate the quality of the tissue at a local level, we performed a parallel analysis of tiles measuring 256*256 pixels throughout the Region of Interest (ROI). We have identified five relevant features to differentiate local quality in whole slide images, as shown in **Supplementary Figure 1c**:

- Focus: We quantify the focus in an image using the Variance of the Laplacian Transform. The Laplacian operator measures the second derivative of an image, highlighting regions of an image with sharp intensity changes. High variance of intensity change, which indicates sharp and smooth changes, is representative of a

normal, in-focus image. Conversely, low variance indicates an image with few sharp edges, typically an out-of-focus image.

- Contrast: We quantify Contrast by measuring the difference between the top 1% of high-value positive pixels and the bottom 1% of low-value positive pixels in each tile. The range must be high enough for good separation of nuclei signal and background.
- Saturation: We measure the percentage of pixels that have a maximum intensity value, which is 255 for 8-bit unsigned integer digital image pixels.
- Artifacts: The main structure of interest in Histology images is usually the nuclei. The morphological open operation, which is an erosion followed by a dilation with the same structuring element for both operations, is used to perform an image opening. If the structuring element or kernel is bigger than the average nuclei size, it highlights dirt and blurry objects that sometimes occur in the images.
- Texture Uniformity: Computing the Gray Level Co-occurrence Matrix (GLCM) calculates how often a pixel with gray-level (grayscale intensity) value "i" occurs horizontally adjacent to a pixel with the value "j". Measuring the uniformity of the pixels allows us to highlight regions with different densities of nuclei. Notably, visceral fat tissue surrounding organs of interest often has a very different texture.

The pipeline was designed for multiplexed fluorescence images, with the algorithm directly applied to the grayscale image of the DAPI fluorescence signal. H&E images are converted to optical density (OD) using a logarithmic transformation before analysis.

A!HistoClouds: The cloud-based digital pathology image annotation and management platform

AI-driven computational pathology diagnosis is an emerging but rapidly developing field. It uses computational algorithms to classify cancer and other diseases, based on the annotated images. Annotating pathological images requires experienced pathologists with years of training. A high-quality annotated image database is the basis for developing AI-based diagnostic solutions, because most successful models are derived from supervised learning. It is important to mark and annotate specific areas/structures/features to describe a disease at the cellular level, and then build and validate the models. Currently, there is no effective "medium" to transfer a pathologist's knowledge and experience to a machine. A!HistoClouds is a cloud-based structural annotation platform designed to enable pathologists to address this gap.

The image viewer (See **Supplementary Figure 2a**) based on the openseadragon software library can visualize DP images with high resolution of 40x objective lens, load image blocks quickly and smoothly, without consuming a lot of device memory and Internet data. The most important basic event functions, such as "zoom", "pan" and "home page", can all be customized using its application programming interface (API) as illustrated in See **Supplementary Figure 2b-c**.

Annotation tool is one of the basic components of A!HistoClouds. The annotation tool provides a ROI management system and has the ability to create an adjustable ROI on top of the image viewer. In other words, when the entire image is moved by the user, the ROI adheres to a specific area on the image. Once the ROI shape is released, it can be fine-tuned. ROI management system refers to a way to easily manipulate and manage many ROIs on the viewer.

A!HistoClouds provides three ROI drawing methods for annotating tasks in the viewer, which can be found in the toolbox button as illustrated in **Supplementary Figure 2d**. They are the freehand drawing, polygonal-dot drawing and brush drawing shown in **Supplementary Figure 2e**. When selecting ROI for further operation, user can click the right-click menu panel. They include labelling, copying, attribute updating and deleting operation. When user click

"More label", ROI can be renamed (default label is “unknown”), and a tag window dialog box will appear for naming choices, as shown in **Supplementary Figure 2f**. Multiple ROI selection is one of the great features love to be used by our pathologist (See Supplementary **Figure 2g**). They first draw many ROIs and then label them at once, which is very helpful for them to save a lot of valuable annotation time.

When pointing the mouse at them, user can easily identify the ROI and related information on the ROI panel as demonstrated in Supplementary Figure 2H. In addition, selected ROI can be hidden and shown easily by click on the "eye" icon at the ROI panel as shown in **Supplementary Figure 2i**. This is a particularly useful feature that allows pathologists to draw ROIs of tissues that may be obscured by another large ROI.

For AI-assisted diagnosis and semi-automatic annotation, the outputs generated by the AI model can be converted into ROI in A!HistoClouds. Therefore, Pathologists can view and modify the ROI annotations using the A!HistoClouds image viewer, and fine-tune accordingly.

Besides, the time spent on annotation is an important measure to understand how easy it is for the pathologist to annotate the entire image slice. They are evidence showing the time spent by pathologists on fully manual annotations and time spent on some fine-tuning (semi-automatic annotations) of the ROI generated based on the AI model. Therefore, A!HistoClouds will automatically record the time spent to draw in each ROI (See **Supplementary Figure 2b**) for performance evaluation.

Hardware and software for model development

We performed AI model training and testing on MATLAB 2021a (MathWorks Inc., USA) with its Deep Learning and Deep Learning, Image Processing and Parallel Computing toolboxes on the Windows 10 X64 operating system. The computer specifications are RAM: 1.0TB, CPU:

Intel(R) Xeon(R) Gold 6242 CPU @ 2.80GHz, and GPU: single NVIDIA Tesla V100-PCIE-16GB.

AI model selection and optimization

Several models of different architectures were initially trained using high-resolution patches (1.12µm/pixel). **Supplementary Figure 3a-c** indicates that NasNet Mobile (macro F1 = 0.68) is slightly inferior to ResNet50 (macro F1 = 0.71) and Vgg16 (macro F1 = 0.71) in terms of macro F1 score, with the differences between ResNet50 and Vgg16 being subtle. Given that ResNet50 has less parameters, it was selected for faster deployment. The models based on ResNet50 structures were trained at four different scales and then applied to test images to compare their performance and optimize the scale factors.

Evaluation of AI model on annotation-level and WSI-level using multiple pathologists' annotations as reference

In this study, the ground truth annotations were reviewed and adjusted by multiple pathologists based on NUH annotations, resulting in different set of annotations, as shown in **Supplementary Figure 4a and b**. Annotations agreed upon senior pathologists were used to assess the models' performances and select the optimal model. Besides, we evaluated the AI model performance using NUH and 9 pathologists' annotations respectively (**Supplementary Figure 4c**). Despite these variations, the model demonstrated superb performance in identifying non-malignant tissues. Inter-observer variations not only exist on annotation level, but also Gleason grading on WSI-level. **Supplementary Figure 4d** demonstrated the Grade Groups (GGs) determined by different pathologists. We assessed the consistency of GG among different pathologists and AI model using Quadratic Weighted Kappa, shown in **Supplementary Figure 4e**.

Three-phase clinical validation of AI-assisted diagnosis

Although the AI model performs well on the image data set, it is imperative to conduct further validation to assess its practical utility in assisting pathologists in real-world application. In our study, we designed a comprehensive three-phase experiment with the objective of comparing the efficacy and efficiency of Gleason Grading through microscopic examination, whole slide image (WSI) examination with and without AI assistance.

For this experiment, we randomly selected 19 slides from the test set, ensuring a representative sample. To establish a reliable ground truth, the Gleason Grade Groups for these slides were independently determined by four senior pathologists. These senior pathologists' assessments were utilized as the reference for calculating the Quadratic Weighted Kappa.

In each phase of the experiment, pathologists meticulously examined the 19 selected slides individually. They assessed and assigned Gleason Scores to each slide while recording the time spent on evaluation. The WSIs were captured at 20× magnification (0.5 µm/pixel) using Akoya Biosciences Vectra Polaris scanner. Phase 3 introduced AI assistance, which encompassed a range of features, including pseudo annotation, tumor percentage, Gleason Pattern percentage, and Gleason Score, all generated by our AI model.

In phase 1, only three pathologists from Singapore participated due to the logistical challenge of shipping glass slides to China. To limit recall bias, we ensured that for each phase and for each pathologist, the order of slide review was intentionally randomized. We also provided comprehensive user guides and pre-experiment training to ensure that all participants were proficient in using A!HistoClouds. To maintain methodological integrity, we implemented a mandatory washout period of at least 20 days between each phase. Additionally, in phases 2 and 3, the filenames of the whole slide images (WSI) were randomly generated, respectively.

Supplementary Tables

Supplementary Table 1 Patient Characteristic Profile of 214 patients included in the study. One patient’s information is missing. Both prostatectomy specimens and biopsy samples were collected from 103 patients, and the other patients provided either prostatectomy specimen or biopsy sample.

Age	Number	Percentage
45–50	1	0.5%
51–60	23	10.7%
61–70	132	61.7%
71–80	49	22.9%
81-90	9	4.2%
**Gleason Score**		
3+3	13	6.1%
3+4	82	38.3%
4+3	58	27.1%
4+4	4	1.9%
3+5	5	2.3%
5+3	2	0.9%
4+5	35	16.4%
5+4	8	3.7%
5+5	7	3.3%

Supplementary Table 2 Configuration of color augmentation All values are subject to ± 5% variance. R, G, B values are first adjusted by addiction/subtraction, then rescaled to [0 1], followed by clipping, in which values below Low_in are mapped to 0 and values above High_in map to 1. Low_in and High_in values apply to all R, G, B channels.

Configuration	**R value**	**G value**	**B value**	**Low_in**	**High_in**
1	-60	-50	-20	0.05	0.95
2	-30	-45	-60	0.05	0.95
3	+35	+70	+35	0.1	0.9

Supplementary Figures

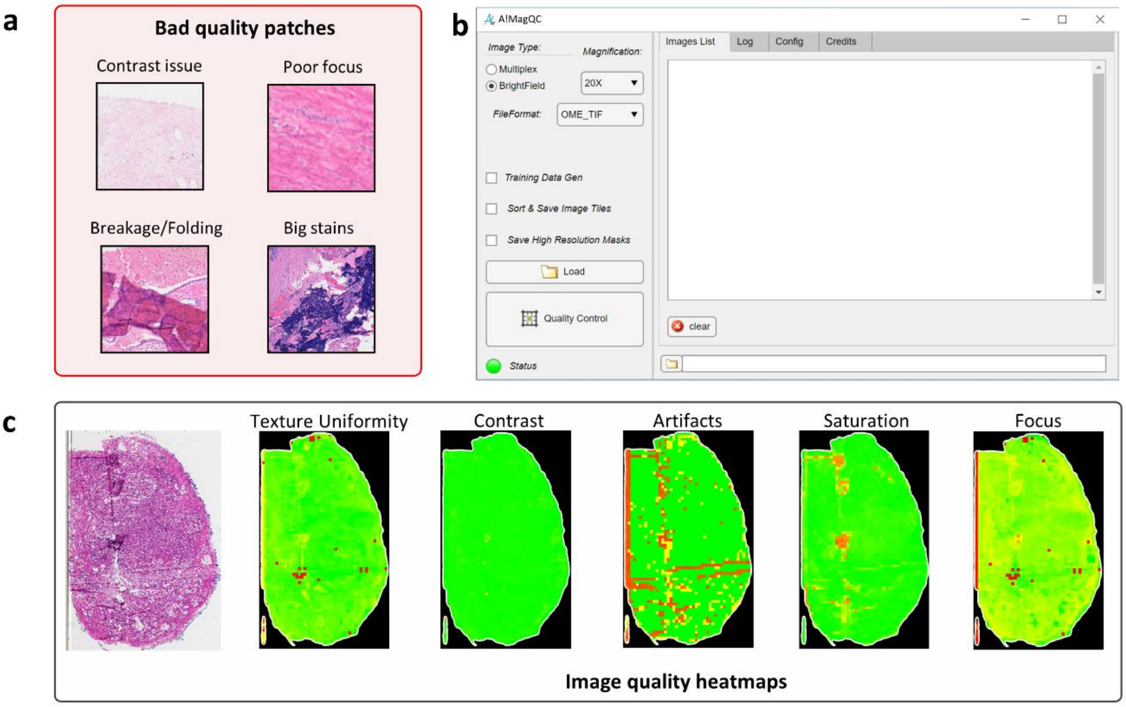

Supplementary Figure 1 Overview of A!MagQC (a) Some examples of common quality issues of histopathological images. (b) User interface of A!MagQC. (c) Heatmap generated by A!MagQC that identify different quality issues. User can easily locate and check the low-quality patches according to the heatmap.

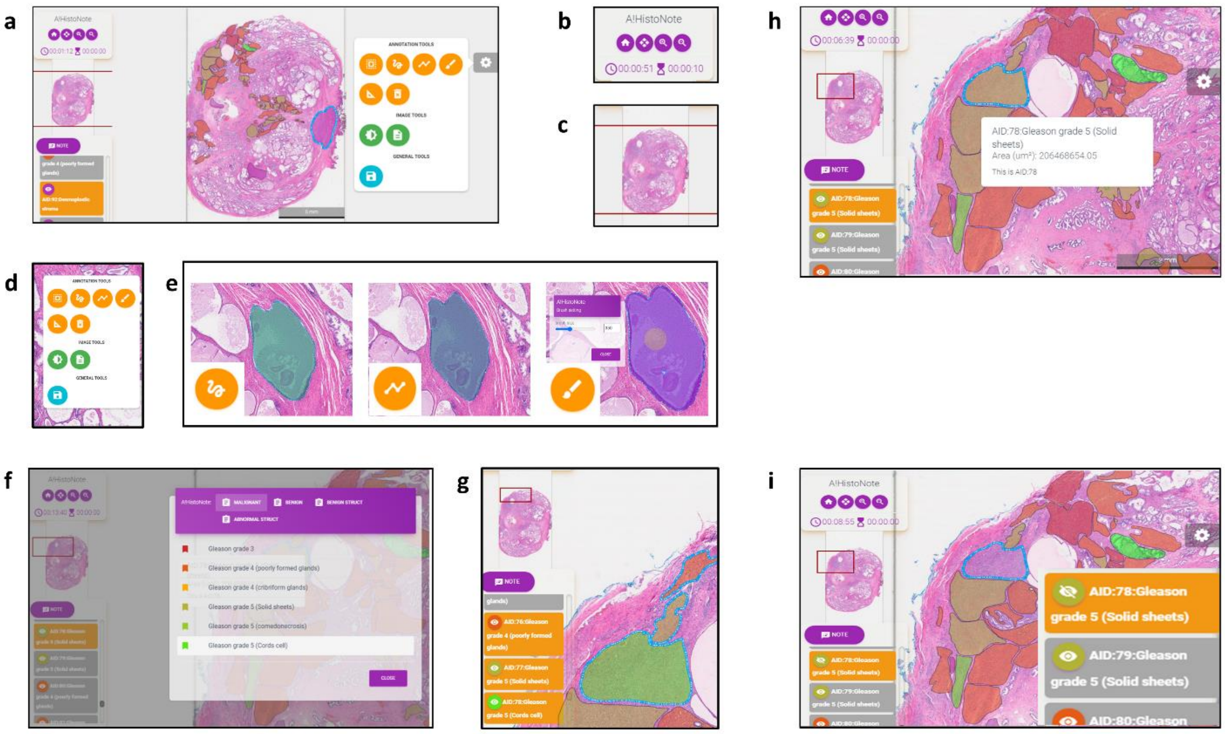

Supplementary Figure 2 Overview of A!HistoClouds A!HistoClouds consists of: (a) image viewer. (b)-(c) timer and basic event function, such as "zoom", "pan" and "home page". (d) toolbox of ROI drawing tools (e) drawing tools: freehand drawing, polygonal-dot drawing and brush drawing. (f) label selection window, (g) annotation panel. (h)-(i) panel of each ROI, where user can easily find the related information of the ROI, and hide the ROI by clicking on the "eye" icon at the ROI panel.

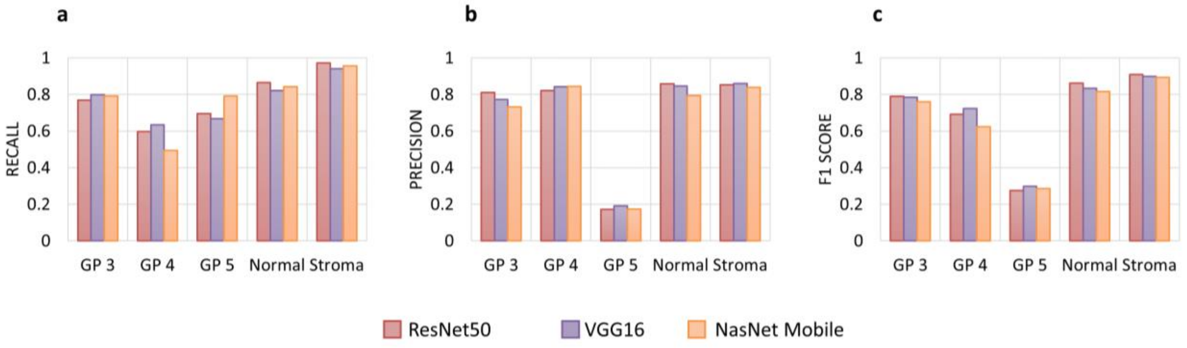

Supplementary Figure 3 Model Selection To select the network architecture, we used high-resolution (1.12μm/px) image patches to train three different models separately and compared their performances. Patch-level performances on test set were shown in (a)-(c). ResNet50 was selected as the preferred model due to its superior performance and smaller network size.

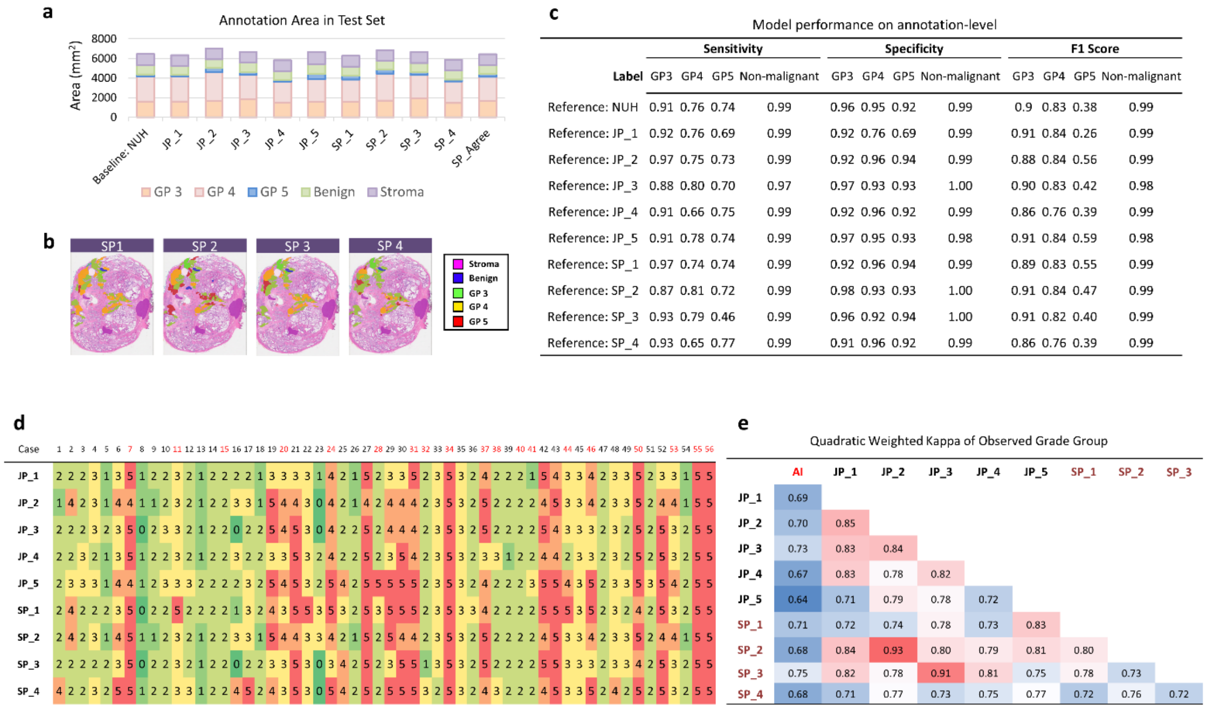

Supplementary Figure 4 Evaluation of Model Performance on Prostatectomy Specimens Using Multiple Pathologists' Annotations The AI model was tested on prostatectomy specimens and compared with annotations made by multiple pathologists to evaluate its performance on both annotation- and WSI-level. The number of annotations made by different pathologists and the agreed upon annotations are presented in (a). Inconsistent annotations made by different pathologists are illustrated in (b), leading to variations in sensitivity, specificity, and F1 score when different standards were applied, as shown in (c). Despite these variations, the model demonstrated superb performance in identifying non-malignant tissues. On the WSI level, GGs determined by different pathologists were summarized in (d). The model achieved a weighted kappa of 0.71 on average with four senior pathologists, while the average weighted kappa among the four pathologists was 0.75, as shown in (e).

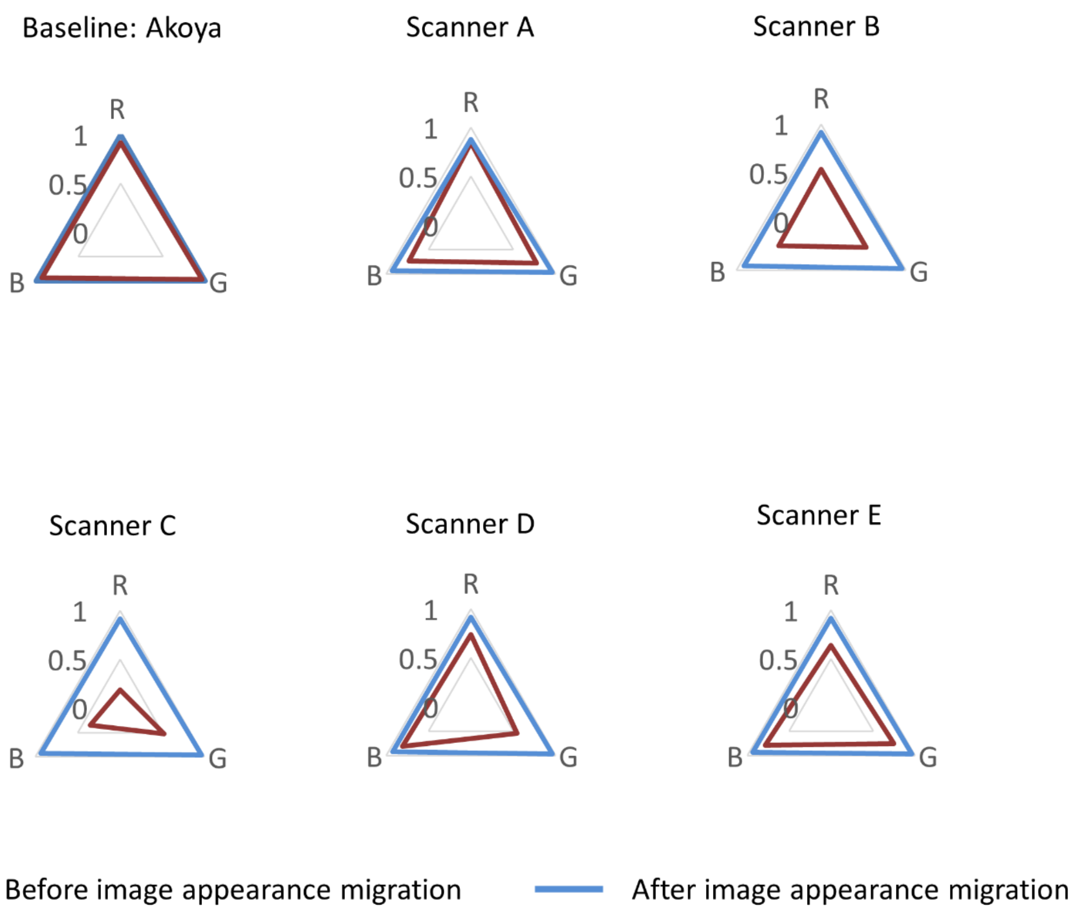

Supplementary Figure 5 Histogram intersection between baseline dataset and other scanner datasets before and after image appearance migration To quantify the effect of image appearance migration, we measured the image similarity before and after migration using histogram intersection of R, G and B channel between baseline and the others. The results showed that migration increased the similarity between baseline and images acquired from other scanners, with almost perfect overlap in histogram intersection for all channels.

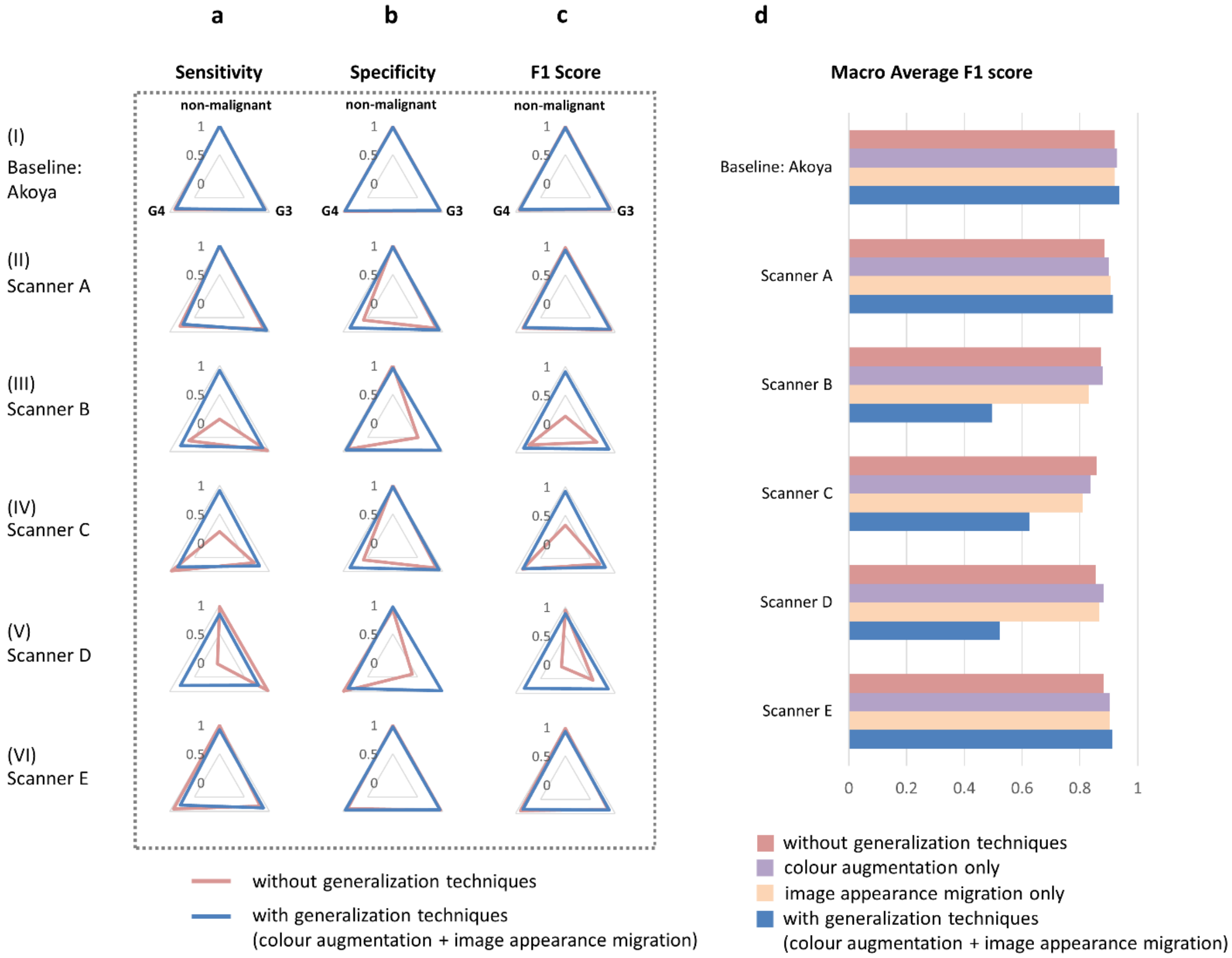

Supplementary Figure 6 Details of model performance across different scanners (a)-(c) Statistical evaluation shows significant improvements in sensitivity, specificity, and F1 score across all classes after applying generalization techniques. (d) The macro average F1 score of each scanner dataset across various generalization techniques is presented. The generalization techniques implemented in this study consist of color augmentation and image appearance migration, and their effects were assessed separately.

Supplementary Notes

Supplementary Note 1 Pseudo code of train-test splitting Considering that prostatectomy specimens are much larger and thus contain more information than biopsies, we used the annotations made by pathologists from NUH on prostatectomy WSIs to train our models. The 187 radical prostatectomy WSIs were split into training and testing sets, whilst the annotated biopsy images were used for testing only. The training and testing set split ratio of prostatectomy WSIs is 7:3 for the number of WSIs, evenly divided to ensure the same ratios of areas for each annotated class in both training and testing since the annotated area of different classes may vary significantly from slide to slide.

Algorithm: Stochastic Search for balanced dataset

Data:

$D = \{(X_i, Y_i)\}_{i=1,\dots,187} \leftarrow$ dataset with 187 images,

$X_i \leftarrow$ prostatectomy specimens image,

$Y_i \leftarrow$ collection of annotations on patch-level,

$C = \{G3, G4, G5, Stroma, Normal\} \leftarrow$ class label sets

Output:

$D_{train} \leftarrow$ training dataset, 70% of D

$D_{test} \leftarrow$ testing dataset, 30% of D

FOUND = *False*

while not FOUND **do**

  Step A:

  Random shuffle the D, let

  $D_{train} \leftarrow \{(X_j, Y_j)\}_{j=1,\dots,132}$

  $D_{test} \leftarrow \{(X_k, Y_k)\}_{k=133,\dots,187}$

  Step B:

  Calculate the number of each class on patch-level in training dataset and test dataset respectively, i.e.

  $N_{train}^{Stroma}, N_{train}^{Normal}, N_{train}^{G3}, N_{train}^{G4}, N_{train}^{G5},$

  $N_{test}^{Stroma}, N_{test}^{Normal}, N_{test}^{G3}, N_{test}^{G4}, N_{test}^{G5}$

  Step C:

  Check the ratio D_{train}/D is 70% ± 5%

  for *Class* in *C* **do**

    $Ratio^{class} = N_{train}^{class} / (N_{train}^{class} + N_{test}^{class})$

    if $65\% \leq Ratio^{class} \leq 75\%$ **then**

      FOUND =True

    else

      FOUND = False

      Break

  end

end

Supplementary Note 2 Pseudo code of voting algorithm During the testing phase, the trained model was applied to test images through a sliding window operation. To ensure that the detection was comprehensive, the window overlap was set at 50%, resulting in the centre box being shared by four consecutive windows. A voting strategy was subsequently employed to determine the label and probability score of each centre box. The final label was determined based on the most frequently occurring label among the four windows. If there was no such label, the final label was chosen based on its higher probability score. The probability score of the center box was then computed as the mean score of the windows corresponding to the final label.

Algorithm: Voting algorithm for each overlapped patch

Input: $D = \{(C_i, S_i)_{i=1,\ldots,4} : C_i \in \{G3, G4, G5, Stroma, Normal\}$, $S_i \in (0,1)$, a collection of label C_i and score S_i for each overlapping patch.

Output: The label C and score S for each overlapped patch.

If $|Set\{C_1, C_2, C_3, C_4\}| = 4$, **then**

- Class: $C = \underset{C}{\mathrm{argmax}} \{S_i : \{C_i, S_i\}\}$
- Score: $S = S_i$

else if Most frequent class C, **then**

- Class: $C = C$
- Score: $S = mean\,(S \mid most\ frequent\ C)$

else

- Class: $C = \underset{C}{\mathrm{argmax}} \{E((S \mid C_i)\}$
- Score: $S = mean\,(S \mid C)$
