# Supplementary material for: A comprehensive AI model development framework for consistent Gleason grading
Source: Commun Med (Lond). 2024 May 9;4:84. doi: 10.1038/s43856-024-00502-1 (PMC11082180; doi:10.1038/s43856-024-00502-1)
Supplement: Supplementary file 3 — Description of Additional Supplementary Files [file 43856_2024_502_MOESM3_ESM.pdf]

Description of Additional Supplementary Files

File name: Supplementary Data
Description: Source data
