# Supplementary material for: A comprehensive AI model development framework for consistent Gleason grading
Source: Commun Med (Lond). 2024 May 9;4:84. doi: 10.1038/s43856-024-00502-1 (PMC11082180; doi:10.1038/s43856-024-00502-1)
Supplement: Supplementary file 5 — Reporting Summary [file 43856_2024_502_MOESM5_ESM.pdf]

nature portfolio

Corresponding author(s): Weimiao Yu

Last updated by author(s): Apr 17, 2024

Reporting Summary

Nature Portfolio wishes to improve the reproducibility of the work that we publish. This form provides structure for consistency and transparency in reporting. For further information on Nature Portfolio policies, see our Editorial Policies and the Editorial Policy Checklist.

Statistics

For all statistical analyses, confirm that the following items are present in the figure legend, table legend, main text, or Methods section.

n/a	Confirmed	
☐	☒	The exact sample size (*n*) for each experimental group/condition, given as a discrete number and unit of measurement
☒	☐	A statement on whether measurements were taken from distinct samples or whether the same sample was measured repeatedly
☐	☒	The statistical test(s) used AND whether they are one- or two-sided<br>*Only common tests should be described solely by name; describe more complex techniques in the Methods section.*
☒	☐	A description of all covariates tested
☒	☐	A description of any assumptions or corrections, such as tests of normality and adjustment for multiple comparisons
☐	☒	A full description of the statistical parameters including central tendency (e.g. means) or other basic estimates (e.g. regression coefficient) AND variation (e.g. standard deviation) or associated estimates of uncertainty (e.g. confidence intervals)
☒	☐	For null hypothesis testing, the test statistic (e.g. *F*, *t*, *r*) with confidence intervals, effect sizes, degrees of freedom and *P* value noted<br>*Give P values as exact values whenever suitable.*
☒	☐	For Bayesian analysis, information on the choice of priors and Markov chain Monte Carlo settings
☒	☐	For hierarchical and complex designs, identification of the appropriate level for tests and full reporting of outcomes
☒	☐	Estimates of effect sizes (e.g. Cohen's *d*, Pearson's *r*), indicating how they were calculated

Our web collection on statistics for biologists contains articles on many of the points above.

Software and code

Policy information about availability of computer code

Data collection	*Provide a description of all commercial, open source and custom code used to collect the data in this study, specifying the version used OR state that no software was used.*
Data analysis	We performed AI model training and testing on MATLAB 2021a (MathWorks Inc., USA) with its Deep Learning and Deep Learning, Image Processing and Parallel Computing toolboxes on the Windows 10 X64 operating system.

For manuscripts utilizing custom algorithms or software that are central to the research but not yet described in published literature, software must be made available to editors and reviewers. We strongly encourage code deposition in a community repository (e.g. GitHub). See the Nature Portfolio guidelines for submitting code & software for further information.

Data

Policy information about availability of data

All manuscripts must include a data availability statement. This statement should provide the following information, where applicable:

- Accession codes, unique identifiers, or web links for publicly available datasets
- A description of any restrictions on data availability
- For clinical datasets or third party data, please ensure that the statement adheres to our policy

The image data set has been published for the Automated Gleason Grading Challenge (AGGC) 2022 as a registered MICCAI 2022 challenge. The dataset can be

downloaded at the challenge website: https://aggc22.grand-challenge.org. The data can be used under an Attribution-NonCommercial-ShareAlike 4.0 International (CC BY-NC-SA 4.0) license. Anyone who uses the data should cite the current article. All source data are available in Supplementary Data.

Human research participants

Policy information about studies involving human research participants and Sex and Gender in Research.

Reporting on sex and gender	The study exclusively utilized images from male patients, aligning with its focus on prostate cancer, a condition inherently specific to males.
Population characteristics	Our study utilized pathological images from 214 male patients diagnosed with prostate cancer in Singapore. Detailed age distribution is available in the Supplementary Materials. No further demographic details were collected, focusing the study on the pathological aspects of prostate cancer within this specific cohort.
Recruitment	Only glass slides in good condition were collected.
Ethics oversight	National Healthcare Group Domain Specific Review Board

Note that full information on the approval of the study protocol must also be provided in the manuscript.

Field-specific reporting

Please select the one below that is the best fit for your research. If you are not sure, read the appropriate sections before making your selection.

☒ Life sciences ☐ Behavioural & social sciences ☐ Ecological, evolutionary & environmental sciences

For a reference copy of the document with all sections, see nature.com/documents/nr-reporting-summary-flat.pdf

Life sciences study design

All studies must disclose on these points even when the disclosure is negative.

Sample size	We collected high-quality glass slides of prostatectomy specimens (prepared during 2017-2019) from National University Hospital Singapore
Data exclusions	No data were excluded.
Replication	Experiment is repeatable with same data and code.
Randomization	Prostatectomy images were stratified-randomly split into training and testing sets
Blinding	All images were anonymized.

Reporting for specific materials, systems and methods

We require information from authors about some types of materials, experimental systems and methods used in many studies. Here, indicate whether each material, system or method listed is relevant to your study. If you are not sure if a list item applies to your research, read the appropriate section before selecting a response.

Materials & experimental systems

n/a	Involved in the study	
☒	☐	Antibodies
☒	☐	Eukaryotic cell lines
☒	☐	Palaeontology and archaeology
☒	☐	Animals and other organisms
☒	☐	Clinical data
☒	☐	Dual use research of concern

Methods

n/a	Involved in the study	
☒	☐	ChIP-seq
☒	☐	Flow cytometry
☒	☐	MRI-based neuroimaging
